# p53, MDM2, eIF4E and EGFR expression in nasopharyngeal carcinoma and their correlation with clinicopathological characteristics and prognosis: A retrospective study

**DOI:** 10.3892/ol.2014.2631

**Published:** 2014-10-24

**Authors:** PENG ZHANG, SONG-KE WU, YING WANG, ZI-XUAN FAN, CHU-RONG LI, MEI FENG, PENG XU, WEI-DONG WANG, JIN-YI LANG

**Affiliations:** 1 Department of Radiation Oncology, Sichuan Provincial Cancer Hospital, Chengdu, Sichuan, 610041, P.R. China; 2 Department of Oncology, Cangxi People’s Hospital, Guangyuan, Sichuan, 618400, P.R. China; 3 Department of Pathology, Sichuan Provincial Cancer Hospital, Chengdu, Sichuan 610041, P.R. China

**Keywords:** p53, MDM2, EGFR, eIF4E, immunohistochemistry, nasopharyngeal carcinoma

## Abstract

In the present study, the expression of p53, mouse double minute 2 homolog (MDM2), eukaryotic translation initiation factor 4E (eIF4E), and epidermal growth factor receptor (EGFR) were investigated in nasopharyngeal carcinoma (NPC), and the correlation between their expression and clinicopathological characteristics and prognosis was analyzed. The medical records of 96 NPC patients who had undergone biopsy prior to radical radiotherapy and chemotherapy between 2005 and 2009 were reviewed, retrospectively. All patients received intensity-modulated radiotherapy with concurrent platinum-based chemotherapy. Patients were followed-up for three years. Streptavidin-peroxidase immunohistochemistry was used to evaluate the expression of p53, MDM2, eIF4E and EGFR in NPC biopsy specimens, and the association between their expression and clinical parameters and survival was analyzed. The p53, MDM2, eIF4E and EGFR expression rates were 65.6% (63/96), 79.16% (76/96), 77.08% (74/96) and 89.5% (86/96), respectively. p53 (χ^2^,20.322; P=0.001) and EGFR (χ^2^,8.337; P=0.005) expression were found to correlate with T stage, whereas MDM2 (χ^2^,16.361; P=0.001) expression was found to correlate with lymph node metastasis. p53 expression was found to inversely correlate with MDM2 expression (r, −3.24; P<0.05). Three-year survival rates were lower in p53-positive (76.2%) patients when compared with p53-negative (93.9%) patients. In addition, three-year survival rates were lower in EGFR-positive (75.8%) patients than in EGFR-negative patients (91.2%). The Cox proportional-hazards regression model revealed that p53 (β,−0.455; χ^2^,5.491; P=0.019) and EGFR (β, 3.93; χ^2^, 11.95; P=0.001) expression were independent prognostic factors. Thus, it was hypothesized that p53 and EGFR expression present potential unfavorable prognostic markers for patients with NPC.

## Introduction

Nasopharyngeal carcinoma (NPC) is the most commonly diagnosed type of head and neck cancer in Southeast Asia, with a reported annual incidence of 30–80 cases per 100,000 individuals in endemic regions (1). The *p53* tumor suppressor gene is one of the most frequently studied genes. p53 mutations or deletions occur with a high frequency in the majority of tumor types and are associated with malignant transformation and tumor progression (2,3). Mouse double minute 2 homolog (MDM2) is an important negative regulator of the p53 pathway and its overexpression has been associated with tumor invasion and metastasis (4). Eukaryotic translation initiation factor 4E (eIF4E), a proto-oncogene, is important in translational regulation and its overexpression selectively increases the mRNA translation of proteins associated with tumor growth, invasion and metastasis. eIF4E overexpression has been identified in various malignant tumors, including cervical, ovarian, esophageal, lung and liver cancer. Previous studies have shown that eIF4E is overexpressed in ~100% of head and neck cancers and has been found to correlate with poor prognosis (5–7). The epidermal growth factor receptor (EGFR), which is encoded by the c-erbB-1 proto-oncogene, is a member of the ErbB family of protein tyrosine kinase receptors and is involved in the regulation of cellular metabolism, growth, migration and differentiation. EGFR is overexpressed in numerous cancer types, including non-small cell lung, breast, prostate and colorectal cancer (8). Therefore, EGFR has become a key target for molecular-based therapies.

The aim of this retrospective study was to examine p53, MDM2, eIF4E and EGFR expression, and their association with clinical characteristics and survival rates in 96 cases of NPC.

## Materials and methods

### Patients and treatment

This study was approved by the Ethics Committee of Sichuan Provincial Cancer Hospital (Chengdu, China). A total of 96 patients with complete medical records, who were treated at Sichuan Provincial Cancer Hospital (Chengdu, China) between 2005 and 2009 were included in the study. The patients consisted of 74 male and 22 female individuals aged between 30 and 77 years (mean age ± standard deviation, 49.9±10.92 years). NPC tissue samples were obtained prior to radiotherapy and chemotherapy using an electronic nasopharyngoscope. All tumor tissue samples were fixed in 10% formalin, embedded in paraffin and cut into 4-μm-thick sections. All patients received intensity-modulated radiotherapy on nasopharyngeal areas, skull basal lesions and positive neck lymph nodes. The following radiotherapy dosages were administered over 28 to 33 sessions: Gross tumor volume (GTV)nx, 66–76 Gy; GTVln, 60–70 Gy; clinical target volume (CTV)1, 60–66 Gy; CTV2, 54–60 Gy; and CTVln, 50–55 Gy. The Cobalt-60 or 6 megavoltage X-ray conventional anterior neck half-field technique was used with a dose of 46–50 Gy for radiotherapy of the lower neck and supraclavicular target regions (9). All patients also underwent concurrent cisplatin-based chemotherapy. Induction chemotherapy regimens included docetaxel (75 mg/m^2^ on day 1) in combination with cisplatin (100 mg/m^2^ on days 1–3) and cisplatin (100 mg/m^2^ on days 1–3) in combination with fluorouracil (750 mg/m^2^ on days 1–4). Concurrent chemotherapy regimens included cisplatin alone (100 mg/m^2^ on days 1–3) and cisplatin (100 mg/m^2^ on days 1–3) in combination with fluorouracil (750 mg/m^2^ on days 1–4). Induction and concurrent chemotherapy comprised of one cycle every 21 days. Supplementary chemotherapy regimens were the same as those used for induction chemotherapy. Written informed consent was obtained from all patients.

### Patient follow-up

All patients were followed-up for between three and six years. During the first year of treatment, patients had one follow-up session every three months. During the first year of treatment patients were followed up every three months. During the second and thurd years following treatment, follow-up was performed every six months. After the third year, follow-up was then performed annually. The follow-up sessions involved indirect nasopharyngoscopy, nasopharyngeal and neck computed tomography/magnetic resonance imaging, abdominal B-mode ultrasonography, chest radiography and blood tests.

### Immunohistochemistry

p53, MDM2, EIF4E and EGFR expression were determined by immunohistochemistry using the histostain-streptavidin peroxidase kit (#95-9943; Invitrogen Life Technologies, Carlsbad, CA, USA) according to the manufacturer’s instructions. Archived paraffin-embedded sections of NPC tumor tissues (4 μm) were deparaffinized and rehydrated. Antigen retrieval was performed using 0.01 M citrate buffer solution (pH 9.0) under pressurized steam for 10 min. The sections were then cooled, washed with phosphate-buffered saline (PBS), blocked with 5% serum and incubated with mouse anti-human monoclonal p53 (1:500 dilution), mouse anti-human monoclonal MDM2 (1:1,000 dilution), rabbit anti-human polyloclonal EIF4E (1:1,000 dilution) and mouse anti-human monoclonal EGFR (1:1,000 dilution) primary antibodies (Abcam, Cambridge, UK) ab1101, ab3110, ab1126, ab131498) overnight at 4°C. After washing with PBS, the tissue samples were incubated with biotinylated goat anti-mouse polyclonal IgG secondary antibody for 60 min at 37°C. Next, the samples were washed with PBS, stained with diaminobenzidine solution, counterstained with hematoxylin, dehydrated and sealed. Positive NPC tissue sections were used as a positive control and tissue sections incubated with PBS instead of primary antibodies were used as negative controls. The percentage of positive cells was quantitated by counting 100 cells in four random microscopic fields (Olympus BH2; Olympus Corporation, Tokoy, Japan). Immunostaining was categorized into four groups according to the percentage of positive cells: (−), <5% positive cells; (+), 5–25% positive cells; (++), 26–50% positive cells; and (+++), ≥51% positive cells. Negative expression was represented by (−) and positive expression by (+) to (+++).

### Statistical analysis

All statistical analyses were performed using SPSS software, version 17.0 (SPSS, Inc., Chicago, IL, USA). Differences in count data were analyzed using the χ^2^ test, and Spearman’s rank correlation test was used for correlational analysis. The Kaplan-Meier estimator was used to calculate survival rates. P<0.05 was considered to indicate a statistically significant difference.

## Results

### Expression of p53, MDM2, EGFR, and eIF4E in NPC

The expression of p53, MDM2, EGFR and eIF4E in NPC was found to be localized in the nucleus. The expression levels of p53, MDM2, EGFR and eIF4E were 65.6% (63/96), 79.16% (76/96), 89.5% (86/96) and 77.08% (74/96), respectively.

### Association between p53, MDM2, EGFR and eIF4E expression and T stage, clinical stage and lymph node metastasis

The expression levels of p53 and EGFR were significantly associated with NPC T stage, according to the 2002 Union for International Cancer Control staging system (Tables I and II) (10). p53 and EGFR expression levels were significantly lower in early T1 and T2 stages than in late T3 and T4 stages (p53; χ^2^,20.322; P=0.001; EGFR: χ^2^, 8.337; P=0.005). The expression levels of p53 and EGFR were also significantly associated with clinical stage (Tables I and II). The expression levels of p53 and EGFR in stage I+II NPC were significantly lower than that in stages III+IV (p53: χ^2^,4.809; P=0.037; EGFR: χ^2^, 15.128; P=0.001). Furthermore, MDM2 expression levels were significantly higher in NPC patients with lymph node metastasis than in those without lymph node metastasis (χ^2^, 16.361; P=0.001) (Table I). However T stage, clinical stage and lymph node metastasis were not associated with eIF4E expression.

### Association between p53 and MDM2 expression in NPC

Spearman’s rank correlational analysis revealed a significant negative correlation between the expression levels of p53 and MDM2 (r, −3.24; P<0.05).

### Association between p53, MDM2, EGFR and eIF4E expression and survival rate

**Figure 1:**
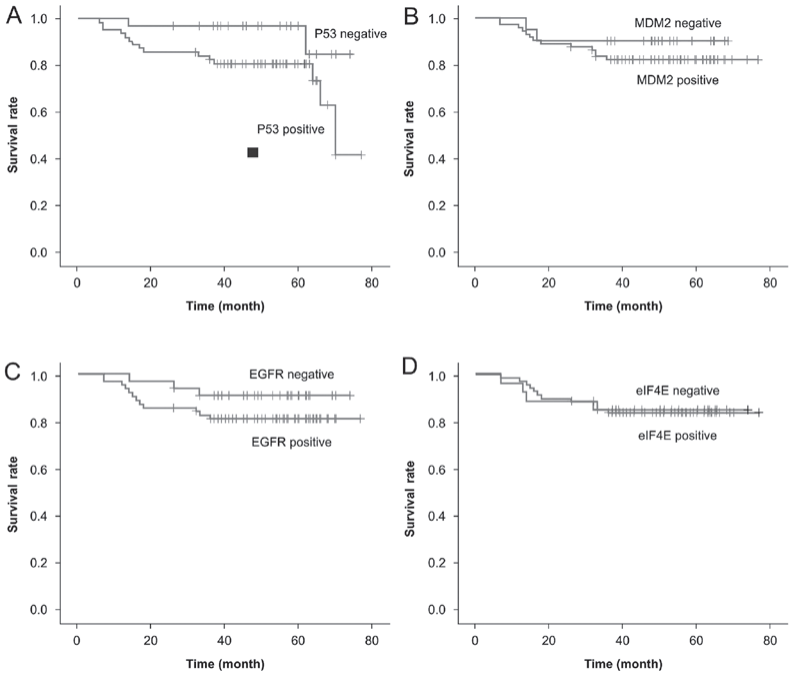
Association between p53, EGFR, MDM2 and eIF4E expression and survival. The Kaplan-Meier method was used to calculate survival rates in 96 nasopharyngeal carcinoma patients. EGFR, epidermal growth factor receptor; MDM2, mouse double minute 2 homolog; eIF4E, eukaryotic translation initiation factor 4E.

The three-year disease-free survival and overall survival (OS) rates for the patient population were 73.9 and 84.4%, respectively. The three-year OS rates were 76.2 and 93.9% in patients with p53-positive and -negative expression, respectively, and 75.8% and 91.2% in patients with EGFR-positive and -negative expression, respectively. Therefore, positive expression of p53 (χ^2^, 4.682; P=0.046) and EGFR (χ^2^, 5.682; P=0.046) was associated with a poor prognosis (Fig. 1). Cox regression model multivariate analysis revealed that p53 (β, −0.455; χ^2^, 5.491; P=0.019) and EGFR (β, 3.93; χ^2^, 11.95; P=0.001) were both independent prognostic factors for survival in NPC. By contrast, MDM2 and eIF4E expression were not associated with survival in NPC.

## Discussion

NPC is a common malignant tumor in China and the standard treatment is radiation therapy. Following radiotherapy, the five-year survival rate is ~40–50% (11). Therefore, it is important to investigate novel adjuvant treatments to improve the five-year survival rates of patients with NPC. With the development of biological therapies for tumors, molecular targeted therapy has become a novel area for the study of tumor therapy. EGFR is expressed in 88–100% of head and neck squamous cell carcinomas, and is important in tumor cell growth, repair and survival. Furthermore, its overexpression often indicates a poor prognosis, early metastasis, chemotherapy resistance or a shorter survival (12–14). Therefore, EGFR has become a key target for cancer gene therapy. In the present study, EGFR was highly expressed in NPC. EGFR-positive expression was observed in 86 out of 96 NPC patients and was found to be associated with aggressive clinical behavior, including advanced T clinical stages. Furthermore, EGFR expression was an independent prognostic factor for survival. A worse survival was observed in patients with positive EGFR expression when compared with patients with negative EGFR expression.

The p53 pathway is one of the most important pathways that regulates the cell cycle. p53 is activated in response to various internal and external stresses and results in an increase in the p21 transcription factor and its translational products. p21 induces G1 cell cycle arrest and DNA repair by inhibiting cyclin-dependent kinase 5 and increasing the expression of the growth arrest and DNA damage inducible genes. When DNA damage cannot be repaired, p53 induces apoptosis by increasing Bcl 2-associated protein X (BAX) expression and the formation of BAX homodimers. Mutations in the p53 gene are one of the most common genetic alterations found in human tumors. Approximately 50% of human tumors exhibit p53 mutations (15–17). In the present study, p53-positive expression was identified in 65.6% of the NPC cases. Positive p53 expression was associated with poor clinical characteristics, including advanced T and clinical stages. Furthermore, p53 expression was an independent prognostic factor of survival. Patients with positive p53 expression exhibited a worse three-year survival rate than those with negative p53 expression.

MDM2 inactivates p53 via direct binding or its E3 ubiquitin ligase activity, which targets p53 for proteasomal degradation. Wild-type p53 is unstable, has a very short half-life and is rapidly degraded by the ubiquitin pathway, whereas mutated p53 is very stable and not easily degraded. In the present study, p53 expression was found to negatively correlate with MDM2 expression. Recent studies have reported that MDM2 expression in malignant tumors is associated with invasion, metastasis, poor prognosis and chemotherapy resistance (18–21). In the present study, high MDM2 expression was identified in NPC (79.16%) and was found to significantly correlate with N stage. MDM2 expression levels were significantly higher in NPC patients with lymph node metastasis than in those without lymph node metastasis. However, MDM2 expression was not associated with T stage, clinical stage or survival in NPC.

eIF4E is a rate-limiting factor in protein synthesis and is distributed throughout the cytoplasm and nucleus in free-floating or multiprotein complex forms in almost all eukaryotic cells. eIF4E expression is regulated by c-myc, RAS and other oncogenes, and its overexpression selectively increases the mRNA translation of proteins associated with tumor growth and invasion, including fibroblast growth factor-2, transforming growth factor-β, platelet-derived growth factor and vascular endothelial growth factor (VEGF). eIF4E overexpression is considered to be a valuable prognostic marker in various tumor types (22,23). In the present study, positive eIF4E expression was present in 74 out of 96 NPC cases (77.08%). However, eIF4E expression was not associated with T stage, clinical stage, lymph node metastasis or survival in NPC.

A complex association has been identified between p53, MDM2 and EGFR expression in a number of human tumors, whereby p53 protein expression was significantly lower and the EGFR and MDM2 expression were higher when compared with that of the normal tissues (24). However, the association between alterations in p53, MDM2, EGFR and the survival of patients with anaplastic astrocytoma or glioblastoma remains controversial (25). In comparison with the initial tumor, recurrent lesions were characterized by a reduced expression of p53 and the number of MDM2 and EGFR positive specimens was reduced. Overexpression and deletion mutations of the EGFR gene, as well as MDM2 overexpression, have been linked to the absence of p53 gene mutations in human glioblastoma multiforme (26). High expression of VEGF and EGFR were independent adverse prognostic factors for long-term outcomes in nonmetastatic NPC independent of clinical stage (27). Mutations of p53 were associated with the overexpression of EGFR and absence of MDM2 in human esophageal carcinomas (28). I*n vivo* studies have revealed that wild-type p53 activates the promoter of EGFR (28). Furthermore, clinical studies have demonstrated that the expression of p53, MDM2 and EGFR are prognostic of human cancers. Numerous studies have reported that the altered expression of these three genes were associated with poor survival and were prognostic of glioblastoma patients (30–37). For example, Ruano *et al* (37) reported that poor outcome in primary glioblastoma multiforme patients is associated with concurrent EGFR and p53 alterations. Furthermore, p53, EGFR and MDM2 are also predictive markers in breast cancer (38), lung cancer (39), blastomatoid pulmonary carcinosarcoma (40), Wilms’ tumor (41), anaplastic thyroid carcinoma (42), bladder cancer (43) and prostate cancer (44). However, p53, MDM2, eIF4E and EGFR have not been previously associated with clinical characteristics in NPC. In this study, the expression of these proteins were detected and their correlation with clinicopathological characteristics and prognosis in NPC was investigated. The results indicated that p53 and EGFR expression correlate with T stage, whereas MDM2 expression correlates with lymph node metastasis. In addition, p53 and EGFR expression were identified as independent prognostic factors in NPC.

## Figures and Tables

**Table I:** Association between p53 and MDM2 expression and the clinical characteristics of 96 nasopharyngeal carcinoma patients.

		p53				MDM2			
							
Clinical characteristics	No. of cases	−	+ to +++	χ^2^ value	P-value	−	+ to +++	χ^2^ value	P-value
T stage				20.322	0.001			1.748	0.215
T1+2	45	5	40			12	23		
T3+4	51	28	23			8	53		
Lymph node metastasis				0.157	0.732			16.361	0.001
No	10	4	6			7	3		
Yes	86	29	57			13	73		
Clinical stage				4.809	0.037			0.976	0.366
I+II	21	3	18			6	15		
III+IV	75	30	45			14	61		

MDM2, mouse double minute 2 homolog.

**Table II:** Association between EGFR and eIF4E expression and the clinical characteristics of 96 nasopharyngeal carcinoma patients.

		EGFR				eIF4E			
							
Clinical characteristics	No. of cases	−	+ to +++	χ^2^ value	P-value	−	+ to +++	χ^2^ value	P-value
T stage				8.337	0.005			4.404	0.051
T1+2	45	9	36			6	39		
T3+4	51	1	50			16	35		
Lymph node metastasis				1.099	0.278			0.317	0.691
No	10	2	8			3	7		
Yes	86	8	78			19	67		
Clinical stage				15.128	0.001			1.651	0.242
I+II	21	7	14			7	14		
III+IV	75	3	72			15	60		

EGFR, epidermal growth factor receptor; eIF4E, eukaryotic translation initiation factor 4E.

## Abbreviations

CTV: clinical target volume
EGFR: epidermal growth factor receptor
eIF4E: eukaryotic translation initiation factor 4E
GTV: gross target volume
MAPK: mitogen-activated protein kinase
MDM2: mouse double minute 2 homolog
NPC: nasopharyngeal carcinoma
PBS: phosphate-buffered saline
PI3K: phosphoinositide 3-kinase
VEGF: vascular endothelial growth factor

## References

[1] Ho FC, Tham IW, Earnest A (2012). Patterns of regional lymph node metastasis of nasopharyngeal carcinoma: a meta-analysis of clinical evidence. BMC Cancer.

[2] Golubovskaya VM, Conway-Dorsey K, Edmiston SN (2009). FAK overexpression and p53 mutations are highly correlated in human breast cancer. Int J Cancer.

[3] Olivier M, Eeles R, Hollstein M (2002). The IARC TP53 database: new online mutation analysis and recommendations to users. Hum Mutat.

[4] Kondo I, Iida S, Takagi Y, Sugihara K (2008). MDM2 mRNA expression in the p53 pathway may predict the potential of invasion and liver metastasis in colorectal cancer. Dis Colon Rectum.

[5] Sunavala-Dossabhoy G, Palaniyandi S, Clark C (2011). Analysis of eIF4E and 4EBP1 mRNAs in head and neck cancer. Laryngoscope.

[6] Singh J, Jayaraj R, Baxi S, Mileva M, Curtin J, Thomas M (2013). An Australian retrospective study to evaluate the prognostic role of p53 and eIF4E cancer markers in patients with head and neck squamous cell carcinoma (HNSCC): study protocol. Asian Pac J Cancer Prev.

[7] Yin X, Kim RH, Sun G, Miller JK, Li BD (2014). Overexpression of eukaryotic initiation factor 4E is correlated with increased risk for systemic dissemination in node-positive breast cancer patients. J Am Coll Surg.

[8] Von Pawel J (2004). Gefitinib (Iressa, ZD1839): a novel targeted approach for the treatment of solid tumors. Bull Cancer.

[9] Feng M, Wang W, Fan Z, Fu B, Li J, Zhang S, Lang J (2013). Tumor volume is an independent prognostic indicator of local control in nasopharyngeal carcinoma patients treated with intensity-modulated radiotherapy. Radiat Oncol.

[10] Greene FL, Page DL (2002). AJCC Cancer Staging Manual.

[11] Lee AW, Lin JC, Ng WT (2012). Current management of nasopharyngeal cancer. Semin Radiat Oncol.

[12] Berg M, Soreide K (2012). EGFR and downstream genetic alterations in KRAS/BRAF and PI3K/AKT pathways in colorectal cancer: implications for targeted therapy. Discov Med.

[13] Baselga J (2001). The EGFR as a target for anticancer therapy - focus on cetuximab. Eur J Cancer.

[14] Nicholson RI, Gee JM, Harper ME (2001). EGFR and cancer prognosis. Eur J Cancer.

[15] Meek DW (2009). Tumour suppression by p53: a role for the DNA damage response?. Nat Rev Cancer.

[16] Chang LJ, Eastman A (2012). Decreased translation of p21 (waf1) mRNA causes attenuated p53 signaling in some p53 wild-type tumors. Cell Cycle.

[17] Seong HA, Ha H (2012). Murine protein serine/threonine kinase 38 activates p53 function through Ser15 phosphorylation. J Biol Chem.

[18] Yin Y, Stephen CW, Luciani MG (2002). p53 stability and activity is regulated by mdm2-mediated induction of alternative p53 translation products. Nat Cell Biol.

[19] Camus S, Ménendez S, Fernandes K (2012). The p53 isoforms are differentially modified by Mdm2. Cell Cycle.

[20] Huang L, Yan Z, Liao X (2011). The p53 inhibitors MDM2/MDMX complex is required for control of p53 activity in vivo. Proc Natl Acad Sci.

[21] Nicholson J, Hupp TR (2010). The molecular dynamics of MDM2. Cell Cycle.

[22] De Benedetti A, Graff JR (2004). eIF-4E expression and its role in malignancies and metastases. Oncogene.

[23] Zimmer SG, DeBenedetti A, Graff JR (2000). Translational control of malignancy: the mRNA cap-binding protein, eIF-4E, as a central regulator of tumor formation, growth, invasion and metastasis. Anticancer Res.

[24] Stark AM, Hugo HH, Witzel P (2003). Age-related expression of p53, Mdm2, EGFR and Msh2 in glioblastoma multiforme. Zentralbl Neurochir.

[25] Ushio Y, Tada K, Shiraishi S (2003). Correlation of molecular genetic analysis of p53, MDM2, p16, PTEN, and EGFR and survival of patients with anaplastic astrocytoma and glioblastoma. Front Biosci.

[26] Halatsch ME, Schmidt U, Unterberg A, Vougioukas VI (2006). Uniform MDM2 overexpression in a panel of glioblastoma multiforme cell lines with divergent EGFR and p53 expression status. Anticancer Res.

[27] Pan J, Tang T, Xu L (2013). Prognostic significance of expression of cyclooxygenase-2, vascular endothelial growth factor, and epidermal growth factor receptor in nasopharyngeal carcinoma. Head Neck.

[28] Esteve A, Lehman T, Jiang W (1993). Correlation of p53 mutations with epidermal growth factor receptor overexpression and absence of mdm2 amplification in human esophageal carcinomas. Mol Carcinog.

[29] Deb SP, Muñoz RM, Brown DR (1994). Wild-type human p53 activates the human epidermal growth factor receptor promoter. Oncogene.

[30] Korkolopoulou P, Christodoulou P, Kouzelis K (1997). MDM2 and p53 expression in gliomas: a multivariate survival analysis including proliferation markers and epidermal growth factor receptor. Br J Cancer.

[31] Harada K, Kurisu K, Tahara H (2000). Telomerase activity in primary and secondary glioblastomas multiforme as a novel molecular tumor marker. J Neurosurg.

[32] Halatsch ME, Schmidt U, Botefur IC (2001). Overexpression of deletion-mutant epidermal growth factor receptor is associated with altered genotoxic stress-provoked p53 mRNA induction in a human glioblastoma cell line. Anticancer Res.

[33] Kleihues P, Ohgaki H (1999). Primary and secondary glioblastomas: from concept to clinical diagnosis. Neuro Oncol.

[34] Stark AM, Hugo HH, Witzel P (2003). Age-related expression of p53, Mdm2, EGFR and Msh2 in glioblastoma multiforme. Zentralbl Neurochir.

[35] Houillier C, Lejeune J, Benouaich-Amiel A (2006). Prognostic impact of molecular markers in a series of 220 primary glioblastomas. Cancer.

[36] Dehais C, Laigle-Donadey F, Marie Y (2006). Prognostic stratification of patients with anaplastic gliomas according to genetic profile. Cancer.

[37] Ruano Y, Ribalta T, de Lope AR (2009). Worse outcome in primary glioblastoma multiforme with concurrent epidermal growth factor receptor and p53 alteration. Am J Clin Path.

[38] Ruiz C, Seibt S, Al Kuraya K (2006). Tissue microarrays for comparing molecular features with proliferation activity in breast cancer. Int J Cancer.

[39] Berghmans T, Mascaux C, Haller A (2008). EGFR, TTF-1 and Mdm2 expression in stage III non-small cell lung cancer: a positive association. Lung Cancer.

[40] Schaefer IM, Sahlmann CO, Overbeck T (2012). Blastomatoid pulmonary carcinosarcoma: report of a case with a review of the literature. BMC Cancer.

[41] Vasei M, Modjtahedi H, Ale-Booyeh O (2009). Amplification and expression of EGFR and ERBB2 in Wilms tumor. Cancer Genet Cytogenet.

[42] Wiseman SM, Masoudi H, Niblock P (2007). Anaplastic thyroid carcinoma: expression profile of targets for therapy offers new insights for disease treatment. Ann Surg Oncol.

[43] Baffa R, Letko J, McClung C, LeNoir J, Vecchione A, Gomella LG (2006). Molecular genetics of bladder cancer: targets for diagnosis and therapy. J Exp Clin Cancer Res.

[44] Bianco R, Caputo R, Caputo R, Damiano V, De Placido S, Ficorella C (2004). Combined targeting of epidermal growth factor receptor and MDM2 by gefitinib and antisense MDM2 cooperatively inhibit hormone-independent prostate cancer. Clin Cancer Res.

